# CT-Guided Preoperative Localization With Coil Placement for Surgical Resection of a Phosphaturic Mesenchymal Tumor: A Case Report and Review of the Literature

**DOI:** 10.7759/cureus.105965

**Published:** 2026-03-27

**Authors:** Tatiana Arroyave Peña, Ricardo Uribe González, Vanessa García Gómez, Emilio Sanín, Alejandro Cardona Palacio, Mauricio Estrada, Miguel Murcia

**Affiliations:** 1 Radiology, Universidad Pontificia Bolivariana, Medellín, COL; 2 Radiology, Hospital Pablo Tobón Uribe, Medellín, COL; 3 Interventional Radiology, Hospital Pablo Tobón Uribe, Medellín, COL; 4 Pathology, Hospital Pablo Tobón Uribe, Medellín, COL; 5 Pathology, Universidad EIA (Escuela de Ingeniería de Antioquia), Medellín, COL; 6 Orthopedic Surgery, Hospital Pablo Tobón Uribe, Medellín, COL

**Keywords:** coil, musculoskeletal, osteomalacia, phosphate, tumor

## Abstract

Phosphaturic mesenchymal tumor (PMT) is a rare neoplasm characterized by excessive secretion of fibroblast growth factor 23 (FGF23) and renal phosphate wasting, leading to osteomalacia and subsequent pathological fractures. These tumors are typically small and slow-growing, which limits their detection. The diagnostic key lies in the use of functional imaging studies such as gallium-68 DOTA-Tyr³-octreotate (68Ga-DOTATATE) positron emission tomography/computed tomography (PET/CT), along with structural imaging modalities such as magnetic resonance imaging (MRI), for the identification of the primary tumor. Definitive treatment consists of complete surgical resection, which normalizes phosphate levels and resolves the patient’s symptoms. We present a diagnostically successful case of a PMT using CT-guided preoperative coil localization before surgical excision, a procedure not previously reported in the literature for these tumors.

## Introduction

Certain typically small and benign neoplasms have the capacity to produce excessive amounts of fibroblast growth factor 23 (FGF23), leading to renal phosphate wasting and decreased vitamin D synthesis. This pathophysiological mechanism results in tumor-induced osteomalacia (TIO), primarily caused by phosphaturic mesenchymal tumors (PMTs) [1,2].

The predominant symptoms of this syndrome include chronic bone pain, progressive muscle weakness, and pathological fractures. A high index of clinical suspicion is required to guide the diagnosis, which remains challenging due to the small size and slow growth rate of these tumors. Functional imaging studies demonstrate high sensitivity and specificity for tumor detection, with gallium-68 DOTA-Tyr³-octreotate (68Ga-DOTATATE) positron emission tomography/computed tomography (PET/CT) being the most prominent modality due to its ability to detect somatostatin receptors expressed by mesenchymal tumors. Contrast-enhanced MRI and CT help define precise tumor localization and anatomical boundaries for appropriate surgical planning and high spatial resolution.

Definitive treatment consists of complete surgical resection, which normalizes FGF23 and phosphate levels, allows bone remineralization, and resolves symptoms. Medical management options with drugs or CT-guided radiofrequency ablation exist for patients who are not surgical candidates, although cure rates are lower compared with surgical treatment [3]. Considering that complete surgical resection is curative, restoring normal phosphate levels and resolving symptoms, our institution performed a CT-guided preoperative coil localization by the interventional radiologist before surgical excision. This procedure has not been previously reported in the literature for these tumors. Using fluoroscopy, the oncologic surgeons were able to easily locate and completely resect the tumor. Ten days after surgery, the patient in this case had normalized phosphate levels and was asymptomatic.

## Case presentation

A 49-year-old male with nephrogenic hypophosphatemia under treatment with phosphorus supplementation, vitamin D3, and calcitriol presented with musculoskeletal symptoms, including lower back pain, left hip pain, and dorsal left foot pain. Laboratory studies revealed decreased serum phosphorus, increased urinary phosphorus excretion, decreased vitamin D levels, and elevated parathyroid hormone (PTH) levels (Table 1). MRI demonstrated a subchondral fracture of the left femoral head with associated bone edema and an ipsilateral hemisacral fracture. Given the presence of pathological fractures in the context of hypophosphatemia, a genetic panel for inherited osteomalacia was ordered, along with whole-body MRI and nuclear medicine studies to rule out TIO secondary to a PMT.

**Table 1:** Laboratory tests This table presents the laboratory values obtained at the beginning of the patient's evaluation (August and September 2025), as well as the values obtained in October when imaging studies were performed. All results showed decreased serum phosphate and vitamin D levels, with elevated parathyroid hormone and urinary phosphate levels. In December 2025, 10 days after surgery, a follow-up was performed in which only serum phosphate was measured, showing complete normalization.

	August 2025	September 2025	October 2025	December 2025	Reference range
Serum phosphate	1.8 mg/dL	1.4 mg/dL	1.7 mg/dL	3.2 mg/dL	2.5–4.5 mg/dL
Urinary phosphate	0.89 g/24 h	1.3 g/24 h	1.8 g/24 h	Not analyzed	0.4–1.3 g/24 h
Vitamin D	9.7 ng/mL	18.7 ng/mL	20 ng/mL	Not analyzed	30–100 ng/mL
Parathyroid hormone (PTH)	51.10 pg/mL	97.7 pg/mL	90 pg/mL	Not analyzed	10–65 pg/mL
Albumin	4.17 g/dL	3.8 g/dL	3.7 g/dL	Not analyzed	4.26–5.05 g/dL

**Figure 1:**
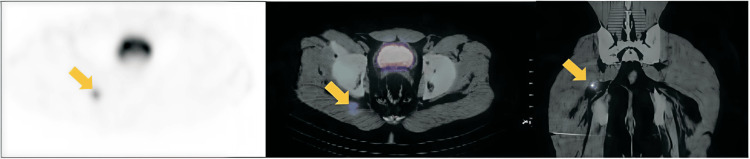
SPECT/CT with 99mTc-HYNIC-TOC Overexpression of somatostatin receptors (Krenning score 3–4) demonstrated by increased radiotracer uptake in a 1.4 × 1.1 cm nodular lesion located between the right gluteus maximus and inferior gemellus muscles (yellow arrows). SPECT: Single-photon emission computed tomography; 99mTc-HYNIC-TOC: Technetium-99m-hydrazinonicotinyl-Tyr^3^-octreotide.

Octreotide scintigraphy with single-photon emission computed tomography (SPECT)/CT showed abnormal radiotracer uptake in the right gluteal region at two and four hours after administration. CT imaging identified a solid nodular lesion with soft-tissue density measuring 1.4 × 1.1 cm located between the right gluteus maximus and inferior gemellus muscles. No additional lesions were detected (Figure 1).

Whole-body MRI demonstrated hyperintense lesions on short tau inversion recovery (STIR) sequences with associated bone marrow edema and contrast enhancement in the distal right humerus, femoral head and neck, left hemisacrum, femoral condyle, and talus, consistent with pathological fractures.

**Figure 2:**
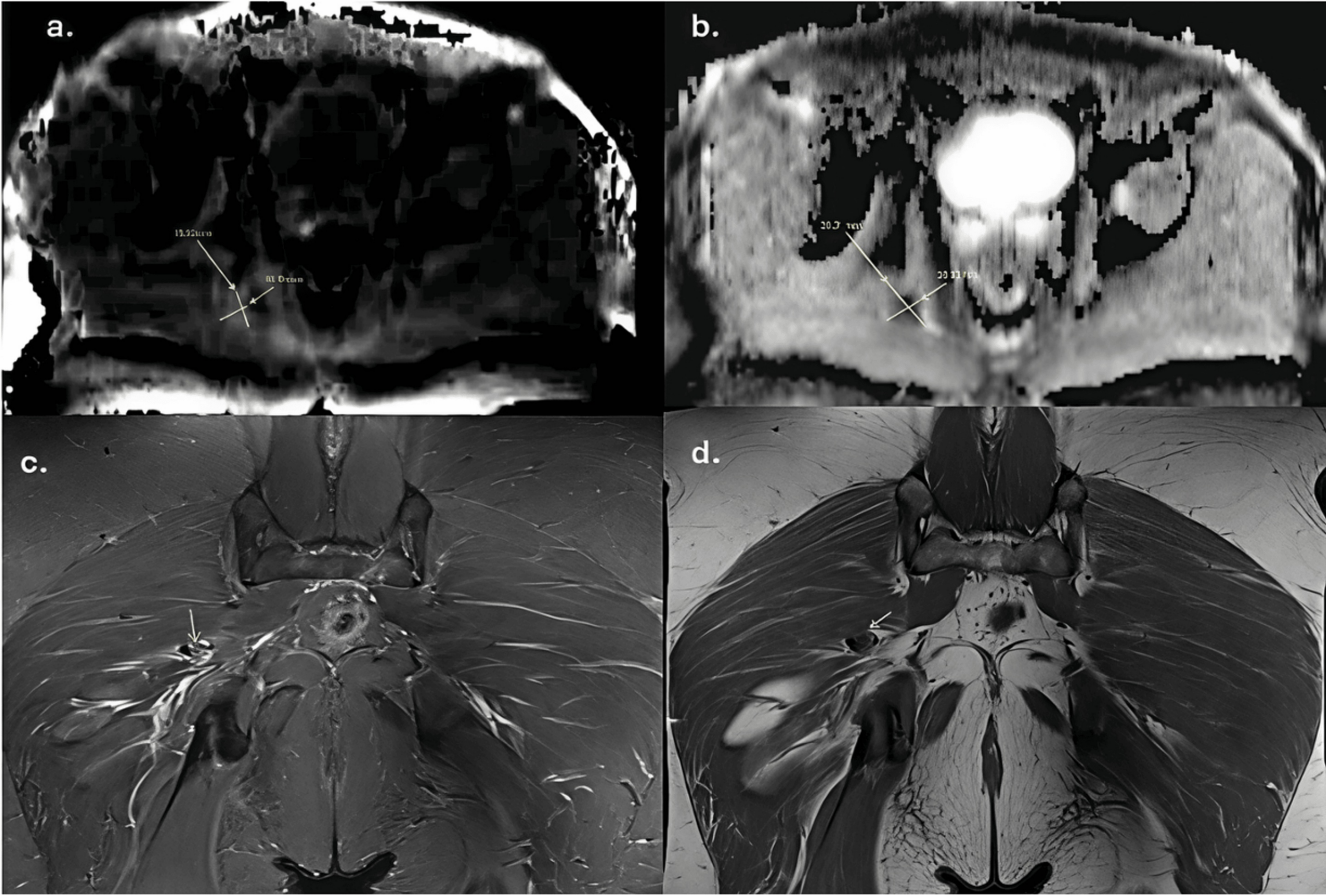
Contrast-enhanced magnetic resonance imaging Nodular lesion (arrows) demonstrating marked diffusion restriction, appearing hyperintense on DWI (a) with low signal values on the ADC map (b), hyperintensity on the STIR sequence corresponding to edema underlying the lesion (c), and isointensity to muscle on T1-weighted imaging (d). DWI: Diffusion-weighted imaging; ADC: Apparent diffusion coefficient; STIR: Short tau inversion recovery.

Additionally, a focal deep right gluteal lesion was identified, appearing hyperintense on STIR, heterogeneous on T1-weighted imaging, with marked post-contrast enhancement and diffusion restriction (Figure 2). A PMT was suspected, and surgical management was planned. Given the lesion’s deep location and small size, preoperative localization was performed.

**Figure 3:**
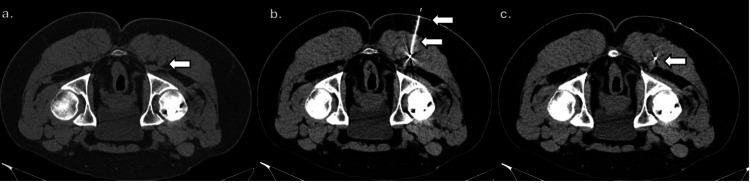
CT-guided preoperative localization with coil placement CT-guided localization of the deep solid tumor within the gluteal musculature (white arrow), isodense to muscle, performed with the patient in the prone position (a); a needle was advanced toward the tumor (double white arrows) (b); and under CT guidance, a platinum coil (white arrow) was deployed along the posterior margin of the lesion (c).

For tumor localization, a platinum coil was inserted adjacent to the posterior margin of the lesion under CT guidance after local anesthesia (Figure 3).

**Figure 4:**
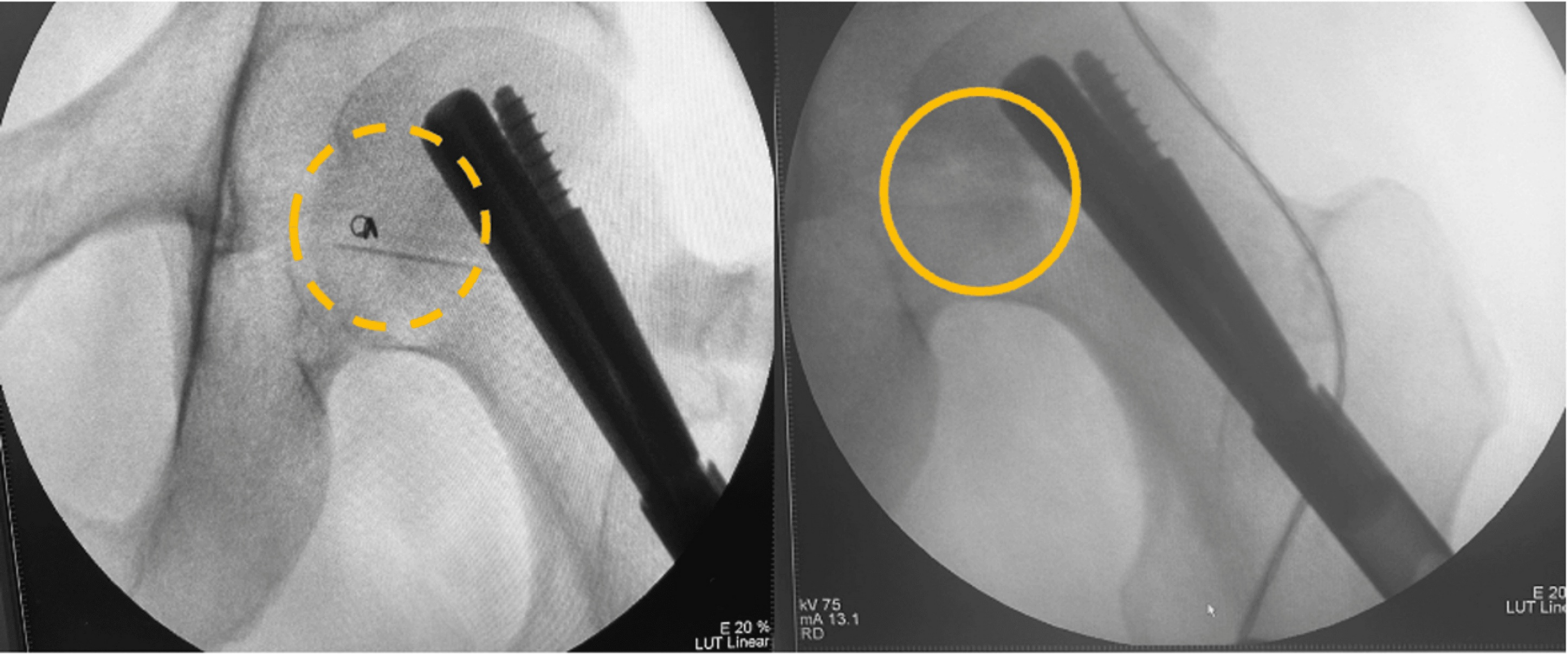
Intraoperative fluoroscopy Intraoperative fluoroscopy demonstrating localization of the coil marking the tumor site (dashed yellow circle), allowing complete excision of the tumor and removal of the marker (continuous yellow circle).

This procedure facilitated intraoperative identification, as the coil was visualized with fluoroscopy, allowing targeted resection of the lesion (Figure 4).

**Figure 5:**
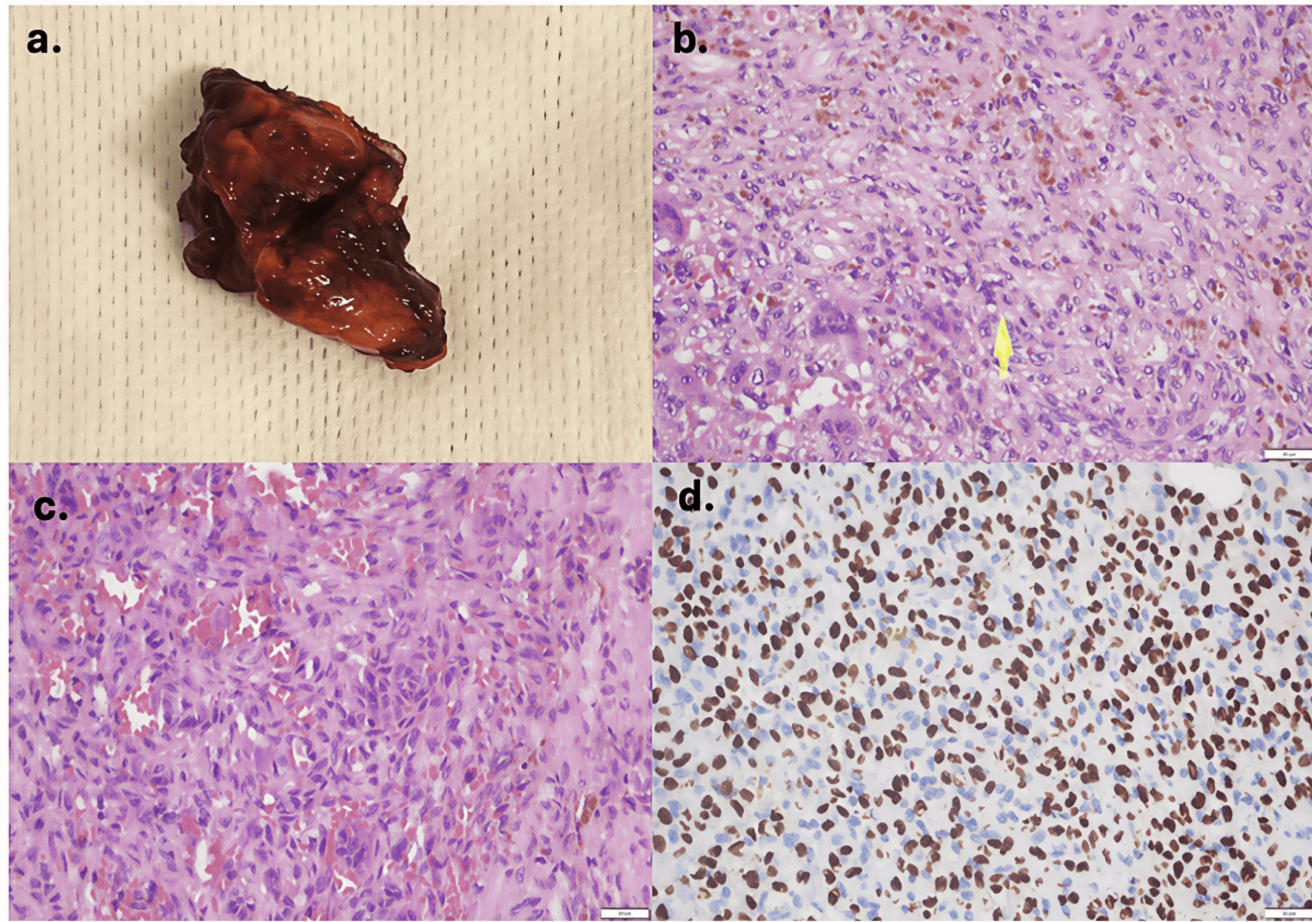
Pathology imaging Gross specimen: small, solid, macrolobulated, and vascularized tumor (a). Spindle-cell mesenchymal proliferation composed of small round to oval nuclei with fine chromatin (yellow arrow) and prominent vasculature (hematoxylin–eosin stain, 40x) (b-c). SATB2 immunohistochemistry (40x) (d). SATB2: Special AT-rich sequence-binding protein 2.

Histopathological examination confirmed a phosphaturic mesenchymal stromal tumor (Figure 5). The tumor was removed, with subsequent resolution of symptoms.

## Discussion

Under normal conditions, osteocytes produce FGF23, which acts on the kidneys by reducing phosphate reabsorption and decreasing vitamin D activation. Vitamin D primarily facilitates intestinal absorption of calcium and phosphate. Excessive production of FGF23 occurs through ectopic, uncontrolled secretion by tumors such as PMTs, leading to tumor-induced osteomalacia [1]. FGF23 decreases phosphate reabsorption in the proximal renal tubules by promoting internalization of sodium-phosphate cotransporters (NaPi 2a and NaPi 2c), resulting in hyperphosphaturia. It also inhibits 1α-hydroxylase, decreasing synthesis of 1,25(OH)₂D (active vitamin D), ultimately impairing bone mineralization [1,2].

Genetically, translocations involving FN1 (fibronectin) and FGFR1 or FGF1 have been described, leading to abnormal activation and overexpression of FGF23, reported in up to 50% of patients in large series from Lee et al. [3].

In pediatric patients, impaired bone mineralization results in rickets, hypotonia, developmental delay, and skeletal deformities [4]. In adults, osteomalacia manifests with progressive bone pain (>90%), gait disturbances, muscle weakness, reduced mobility, and pathological or insufficiency fractures (69%-80%). Secondary or tertiary hyperparathyroidism may develop in chronic cases [1,4-6].

TIO is rare, with approximately 1,000 cases reported worldwide [6]. With nonspecific symptoms such as those described, it predominantly affects adults in approximately 80% of cases, of whom more than half (56%) are male, with a mean age of 49 years [7]. Diagnosis is delayed in up to 95% of cases, sometimes by as much as 6.7 years [8].

PMTs most commonly arise in bone and soft tissues. More than half arise in the lower extremities, most commonly in the femur (42%), pelvis (21.7%), and tibia (12.3%), as well as in soft tissues (24.1% in the thighs and 21% in the feet). Fewer than 10% of tumors are located in the upper extremities and trunk, 24% occur in the craniofacial region, and visceral metastases are even less frequent, most commonly involving the lungs [1,7,9]. They are small, deep-seated, non-palpable, and slow-growing. Approximately 93% are benign, and 6%-7% are malignant. The most common subtype (70%-84.9%) is the mixed connective tissue variant. Microscopically, these tumors are characterized by low-grade spindle cells with a prominent vascular component and osteoclast-like multinucleated giant cells within a variable chondromyxoid or osteoid matrix [7,10].

Diagnosis begins with biochemical findings of hypophosphatemia, hyperphosphaturia, low active vitamin D levels, elevated alkaline phosphatase, and elevated FGF23 levels (>96%) [7]. Magnetic resonance imaging (MRI) with contrast is considered the imaging gold standard for anatomical evaluation, with reported sensitivity of 94%-100% [7]. Lesions are typically iso- to hypointense on T1-weighted imaging and hyperintense on T2-weighted imaging, show strong contrast enhancement, and may demonstrate diffusion restriction. STIR is the sequence of choice because of its superior contrast resolution, enabling accurate tumor delineation by demonstrating high signal intensity relative to adjacent soft tissues [11].

CT is useful for surgical planning and evaluation of the tumor matrix and bone involvement. It has the advantage of allowing evaluation of the tumor matrix, which is most commonly punctate, amorphous, or ground glass in appearance, and provides high sensitivity for detecting bone lesions, which are typically osteolytic with a narrow zone of transition [7,11]. In this case, CT-guided coil localization was used before surgical excision, a technique not previously described for PMTs. This approach enabled precise intraoperative localization and successful complete resection.

Once the lesion was identified on MRI, given its depth and the previously described characteristics of these small, non-palpable tumors, CT-guided preoperative localization was performed before surgical resection. The procedure was carried out with the patient in the prone position. A non-contrast CT scan was obtained to localize the tumor, which was then accessed using a 21-gauge needle, followed by placement of a Hilal 18-3-3 platinum coil along the posterior margin of the lesion (Figure 2).

With this localization, the orthopedic oncology team identified the coil intraoperatively using fluoroscopic image guidance (Figure 3), allowing determination of the surgical approach and successful complete resection of the tumor (Figure 4).

Functional imaging plays an important role in tumor detection. Its primary objective is to identify metabolic activity and the expression of specific receptors. The modality with the highest sensitivity and accuracy is 68Ga-DOTATATE PET/CT, which demonstrates high affinity for somatostatin receptors, high spatial resolution, short acquisition time, and low radiation dose due to the physical properties of gallium-68, achieving sensitivity close to 100% [12].

Fludeoxyglucose F18 (FDG) PET/CT is more widely available but has lower specificity due to a high rate of false-positive results, as it detects increased glucose metabolism in processes with high glycolytic activity, such as inflammation or fractures. Its sensitivity ranges from 67% for soft-tissue tumors to 85.7% for bone tumors, with a specificity of approximately 60% [4,7].

Octreotide SPECT/CT is one of the most established techniques because of its accessibility; however, it involves higher radiation doses, longer acquisition times, and lower spatial resolution compared with PET/CT, with reported sensitivity ranging from 36.3% to 63% [12]. Like octreotide, 68Ga-DOTATATE is a somatostatin receptor antagonist that, upon binding, leads to receptor internalization and intracellular accumulation of the radiotracer within tumor cells. Breer et al. reported that PMTs predominantly express somatostatin receptor subtype 2, for which DOTATATE has greater affinity compared with octreotide, which preferentially binds subtype 5 receptors [13]. This difference explains the greater sensitivity and diagnostic accuracy of 68Ga-DOTATATE PET/CT compared with octreotide SPECT/CT.

The literature supports that, in addition to these functional imaging studies, anatomical imaging should be performed in all patients to confirm tumor localization before resection and during follow-up after normalization of serum phosphate levels to confirm cure [12].

Complete surgical resection with negative margins is curative in approximately 90% of cases [14]. Recurrence and metastasis rates are low (<10%) [15,16]. Postoperatively, serum phosphate and FGF23 normalize within days to weeks, and bone remineralization may take up to one year. When tumors are inoperable, minimally invasive procedures such as radiofrequency ablation, cryoablation, or stereotactic radiotherapy may be considered [17,18]. Medical therapy includes phosphate and calcitriol supplementation, which are poorly tolerated due to their unpleasant taste and associated gastrointestinal side effects [19]. Since 2020, burosumab, a monoclonal antibody against FGF23, has been approved and significantly improves biochemical and clinical outcomes with low rates of adverse effects [20].

A literature review of the most representative cases, including diagnostic methodology and treatment, is presented in Table 2. In most cases, the main diagnostic marker was elevated FGF23 levels; however, this laboratory test is not available in our country. Most cases were treated with surgical resection [3]. Cases treated with CT-guided radiofrequency ablation showed recurrence of the disease [21], whereas inoperable cases with high surgical risk were treated with burosumab [22].

**Table 2:** Literature review of the most representative cases Boyle et al. reported cases in which the primary tumors were mostly located in the lower limbs, and diagnosis was performed using 68Ga-DOTATATE PET/CT; however, recurrence occurred after treatment with radiofrequency ablation. Lee et al. presented nearly 15 cases (only representative cases shown in the table) with favorable outcomes after surgical resection. Cadiou et al. described inoperable cases that required treatment with the monoclonal antibody (burosumab). 68 Ga-DOTATATE: Gallium-68 DOTA-Tyr³-octreotate; PET/CT: Positron emission tomography/computed tomography; FGF23: Fibroblast growth factor 23.

Age, sex	Tumor localization	Diagnosis	Treatment	Author
40, female	Left femoral head	Low phosphate levels and elevated FGF23, 68 Ga-DOTATATE PET/CT	CT-guided radiofrequency ablation	Boyle et al., 2022 [21]
36, female	Left acetabulum	Vertebral insufficiency fractures and severe hypophosphatemia, 68 Ga-DOTATATE PET/CT	Two CT-guided radiofrequency ablations because of recurrence	Boyle et al., 2022 [21]
60, female	Left femur	Chronic pain, 68 Ga-DOTATATE PET/CT	Two CT-guided radiofrequency ablations because of recurrence	Boyle et al., 2022 [21]
44, male	Thigh	Elevated FGF23	Surgical resection	Lee et al., 2016 [3]
55, male	Ankle	Elevated FGF23	Surgical resection	Lee et al., 2016 [3]
61, male	Glute	Elevated FGF23	Surgical resection	Lee et al., 2016 [3]
Adult, male	Femoral head	Limited resection due to high risk of bone necrosis	Burosumab	Cadiou et al., 2025 [22]
Adult, male	Spinal	Inoperable tumor discovered following a previous surgery	Burosumab	Cadiou et al., 2025 [22]
Adult, male	Spinal canal	Limited resection due to high risk of neurological damage	Burosumab	Cadiou et al., 2025 [22]
Adult, female	Lung (metastases)	Primary tumor of the tibia resected, with metastases	Burosumab	Cadiou et al., 2025 [22]
Adult, female	Lung (metastases)	Primary tumor of the patella resected, with metastases	Burosumab	Cadiou et al., 2025 [22]

## Conclusions

Diagnosis of PMT requires a high index of suspicion in patients presenting with osteomuscular symptoms and unexplained pathological fractures associated with hypophosphatemia. Functional imaging, such as 68Ga-DOTATATE PET/CT, is essential for tumor detection, complemented by anatomical imaging such as MRI for precise localization. Complete surgical resection is frequently curative. Multidisciplinary collaboration among orthopedics, oncology, endocrinology, pathology, and radiology is crucial to reduce diagnostic delay and morbidity.

## References

[1] Dahir K, Zanchetta MB, Stanciu I (2021). Diagnosis and management of tumor-induced osteomalacia: perspectives from clinical experience. J Endocr Soc.

[2] Tang T, Jin H, Yang Y (2023). Imaging manifestations of phosphaturic mesenchymal tumors: a description of two cases. Quant Imaging Med Surg.

[3] Lee JC, Su SY, Changou CA (2016). Characterization of FN1-FGFR1 and novel FN1-FGF1 fusion genes in a large series of phosphaturic mesenchymal tumors. Mod Pathol.

[4] Hussein MA, Cafarelli FP, Paparella MT, Rennie WJ, Guglielmi G (2021). Phosphaturic mesenchymal tumors: radiological aspects and suggested imaging pathway. Radiol Med.

[5] Avila NA, Skarulis M, Rubino DM, Doppman JL (1996). Oncogenic osteomalacia: lesion detection by MR skeletal survey. AJR Am J Roentgenol.

[6] Florenzano P, Hartley IR, Jimenez M, Roszko K, Gafni RI, Collins MT (2021). Tumor-induced osteomalacia. Calcif Tissue Int.

[7] Maier JP, Krapohl MA, Herget GW (2025). Surgical treatment as a key determinant of outcome in phosphaturic mesenchymal tumors of the bone and soft tissue: a systematic review and case series. EFORT Open Rev.

[8] Jiang Y, Xia WB, Xing XP (2012). Tumor-induced osteomalacia: an important cause of adult-onset hypophosphatemic osteomalacia in China: report of 39 cases and review of the literature. J Bone Miner Res.

[9] Jiang Y, Hou G, Cheng W (2020). Performance of 68Ga-DOTA-SST PET/CT, octreoscan SPECT/CT and 18F-FDG PET/CT in the detection of culprit tumors causing osteomalacia: a meta-analysis. Nucl Med Commun.

[10] Chong WH, Andreopoulou P, Chen CC (2013). Tumor localization and biochemical response to cure in tumor-induced osteomalacia. J Bone Miner Res.

[11] Broski SM, Folpe AL, Wenger DE (2019). Imaging features of phosphaturic mesenchymal tumors. Skeletal Radiol.

[12] El-Maouche D, Sadowski SM, Papadakis GZ (2016). (68)Ga-DOTATATE for tumor localization in tumor-induced osteomalacia. J Clin Endocrinol Metab.

[13] Breer S, Brunkhorst T, Beil FT (2014). 68Ga DOTA-TATE PET/CT allows tumor localization in patients with tumor-induced osteomalacia but negative 111In-octreotide SPECT/CT. Bone.

[14] Barrera CA, Karp J, Kearns C, Rhodes NG (2023). Phosphaturic mesenchymal tumor. Radiographics.

[15] Shi Z, Deng Y, Li X, Li Y, Cao D, Coossa VS (2018). CT and MR imaging features in phosphaturic mesenchymal tumor-mixed connective tissue: a case report. Oncol Lett.

[16] Li X, Jiang Y, Huo L (2020). Nonremission and recurrent tumor-induced osteomalacia: a retrospective study. J Bone Miner Res.

[17] Mishra SK, Kuchay MS, Sen IB, Garg A, Baijal SS, Mithal A (2019). Successful management of tumor-induced osteomalacia with radiofrequency ablation: a case series. JBMR Plus.

[18] Vásquez A, Ortegón JDC, Bermúdez LMO, Moreno A, Gaitán KC (2025). Osseous abnormalities associated with phosphaturic mesenchymal tumor. Radiol Imaging Cancer.

[19] Chong WH, Molinolo AA, Chen CC, Collins MT (2011). Tumor-induced osteomalacia. Endocr Relat Cancer.

[20] Lamb YN (2018). Burosumab: first global approval. Drugs.

[21] Boyle V, Pinto C, Hoon D (2022). Radiofrequency ablation, a potential cure for tumour-induced osteomalacia: case reports of short- and long-term outcomes [PREPRINT].

[22] Cadiou S, Chapurlat R, Couture G (2025). Real-world efficacy and safety of burosumab in tumor-induced osteomalacia: case series from an early access program. JBMR Plus.

